# The National Comprehensive Governance for epilepsy prevention and control in China

**DOI:** 10.1002/epi4.12554

**Published:** 2021-11-08

**Authors:** Shichuo Li, Yuping Wang, Wenzhi Wang, Dong Zhou, Hui Zhang, Lirong Duan, Ding Ding, Yuwu Jiang, Yi Wang, Xiaoyan Liu, Zhen Hong

**Affiliations:** ^1^ China Association Against Epilepsy Beijing China; ^2^ Department of Neurology Xuanwu Hospital Capital Medical University Beijing China; ^3^ Beijing Neurosurgical Institute Beijing China; ^4^ Department of Neurology West China Hospital Sichuan University Chengdu China; ^5^ Neurology Department Huashan Hospital Fudan University Shanghai China; ^6^ Department of Pediatrics Peking University First Hospital Beijing China; ^7^ Department of Neurology Children's Hospital of Fudan University Shanghai China

**Keywords:** China, epilepsy, Governance, prevention and control

## Abstract

This article describes “the national comprehensive governance for epilepsy prevention and control in China” as an example of a strategic working program for the prevention and control of epilepsy at a national level. The comprehensive governance includes 10 key elements: epilepsy centers' consortium and three tier diagnosis/treatment network; rural and urban community projects on epilepsy care; awareness, publicity, and education; big data platform; development of telemedicine; team and capacity building; strengthening epilepsy research; developing and strengthening partnership; coordination with government; and international collaboration.

**Key Points**

- Leadership and governance are critical levers to address the challenges faced by people with epilepsy and to improve epilepsy care overall
- The governing body for epilepsy control in China is the China Association Against Epilepsy under the leadership of government
- There are 10 key ingredients of the comprehensive governance for epilepsy control in China
- The model of the governance for epilepsy control in China may serve as a blueprint for other LMIC

## 1 INTRODUCTION

Epilepsy is one of the most common neurological diseases, affecting around 50 million people of all ages, genders, ethnic backgrounds, and geographic locations worldwide. The risk of premature death in people with epilepsy (PWE) is up to three times that of the general population. In all settings, PWE always face unmet needs in access to education, employment, residential and community services, and appropriate and affordable healthcare.^1^

The global epilepsy report (GER) “EPILEPPSY – A public health imperative” published by the World Health Organization (WHO), in 2019,^1^ emphasizes that leadership and governance are critical levers to address the challenges faced by PWE and to improve epilepsy care overall. Likewise, it has been stressed that “to promote prevention and control of epilepsy, we need coordinated actions at the national level to strengthen effective leadership and governance.”^2^

In this article, we highlight the role of comprehensive “governance” as a key policy for prevention and control of epilepsy at the national level, describing the approach taken by China as an example.

## 2 BACKGROUND

Over the centuries, epilepsy has been treated with “Traditional Chinese Medicine” and other folk therapies in China. The introduction of modern medicine, including surgical treatment, in the 20th century led to a major improvement in epilepsy care.^3^

In 1983, the first nationwide epidemiological survey for neurological disorders including epilepsy was carried out in six urban and 22 rural areas covering a population of 300 000 individuals. As a part of the Global Campaign Against Epilepsy (GCAE) Demonstration Project (DP) funded by the WHO, the International League Against Epilepsy (ILAE), and the International Bureau for Epilepsy (IBE), another epidemiological survey was conducted in 2000 in rural areas of five provinces. The results of these surveys showed a prevalence of 0.7% and an annual incidence rate of 35/100 000.^4^, ^5^, ^6^ According to the results of the 7th National census, the population in China is 1.41 billion,^7^ it can be estimated that there are near 10 million PWE living in China, including approximately 6 million people with active epilepsy and around 400 000 new cases each year. About two‐thirds of PWE in China are estimated not to be receiving treatment, or to receive a treatment that is either inadequate or inappropriate.^8^ This huge treatment gap is mainly driven by deficiencies in the delivery of healthcare and by the social discrimination against PWE as a result of cultural beliefs. Enacted stigma is almost universal and affects not only individuals with epilepsy, but also their families.^9^

Since the establishment of the China Association Against Epilepsy (CAAE) in 2005, there has been major progress in epilepsy care in China, with a positive impact on prevention, diagnosis, and treatment. Addressing the needs of about 10 million PWE in the nation is a big challenge. In the sections below, we describe the governance measures that we have planned, most of which are already implemented.

## 3 THE GOVERNING BODY

The prevention and control of epilepsy are inevitably under the responsibility of the government. Because of the wide range of health issues that need to be tackled, the government cannot take detailed concrete leadership for all actions against epilepsy. Therefore, in its position as a national nongovernmental organization and as a chapter of the ILAE and IBE, the CAAE has taken a leading role in these actions. Based on this, we define as “governing body” for epilepsy prevention and control in China: “the China Association Against Epilepsy, including the provincial associations against epilepsy, under the leadership of the government and in collaboration with other relevant professional organizations.” The CAAE's organizational structure is shown in Table 1.

**TABLE 1:** Organizational structure of CAAE

As “governing body” for epilepsy prevention and control in China, CAAE has organizational structure as follows:	
Membership	10 687
Board Membership	255 (standing members 81)
Leading Group	President 1
	Vice presidents 11
	Secretary General 1
Provincial Associations Against Epilepsy	28 of 31 provinces/municipalities
Professional Branch	CAAE EEG & Neurophysiology Branch (CENB)
Special Topic Oriented and Management Committees	1. CAAE Bureau for Epilepsy (CBE)
	2. CAAE Youth Commission (CYC)
	3. CAAE Commission on Standardized Development of Epilepsy Centers (CSDEC)
	4. CAAE Epilepsy Co‐morbidity Commission (CECC)
	5. CAAE Tuberous Sclerosis Complex (TSC) Commission (CTSCC)
	6. CAAE SEEG & Brain Mapping Commission (CSBMC)
	7. CAAE Precision Medicine & Medication Adverse Event Monitoring Commission (CPMMAC)
	8. CAAE Neuromodulation Commission (CNMC)
	9. CAAE Management Committee for the TAN QIFU Epilepsy Surgery Development Funds (CMTESF)
	10. CAAE Innovation & Translation Commission (CITC)
	11. CAAE Commission on Epilepsy Medicines Therapy (CCEMT)
	12. CAAE Ketogenic Diet Commission (CKDC)
	13. CAAE Epilepsy community management Committee (CECMC)
Official Journals	Journal of Epilepsy (Chinese);
	Acta Epileptologica (English)

Abbreviations: CAAE, China Association Against Epilepsy; EEG, electroencephalography.

## 4 THE ESSENTIAL ELEMENTS OF A COMPREHENSIVE GOVERNANCE

Based on our practice established over the past 17 years and plans made for the years to come, we identified 10 key components of the comprehensive governance.

### 4.1 Epilepsy centers' consortium and three tier diagnosis/treatment network

In last two decades, various epilepsy centers have been gradually developed to provide different levels of service for PWE in China. The CAAE performed a nationwide investigation of epilepsy centers in 2017‐2018, covering 31 provinces, autonomous regions, and municipalities. The investigation identified a total of 358 “epilepsy centers” in China, of which 338 (94.4%) were in public hospitals and 20 (5.6%) in private hospitals. Of these hospitals, 333 (93.0%) were tertiary grade, and 25 (7.0%) were secondary grade.^10^

By referring to the 2001 guidelines of the US National Association of Epilepsy Centers (NAEC),^11^ we established grading standards for a three‐tier system of epilepsy centers and classified the 358 “epilepsy centers” into three categories, namely Tertiary‐level Comprehensive Epilepsy Center (TCEC), Secondary‐level Epilepsy Center (SEC), and Primary‐level Epilepsy Service Facility (PESF).^12^

In 2018 and 2019, we completed an accreditation procedure for TCECs. Among 69 centers that applied for accreditation, 31 were ratified as TCECs. These centers are distributed in most provinces' capital cities and municipalities and can serve as high‐level referral centers for epilepsy diagnosis and treatment. The accreditation procedures for SECs and PESFs have initiated in 2021 by the provincial level associations against epilepsy using the grading system set up by the CAAE.

We are establishing a “Consortium of Epilepsy Centers” in China to promote coordination, collaboration, and information sharing among TCECs, with the aim of establishing a nationwide network of epilepsy centers with high‐level expertise in the diagnosis and treatment of epilepsy.

**FIGURE 1:**
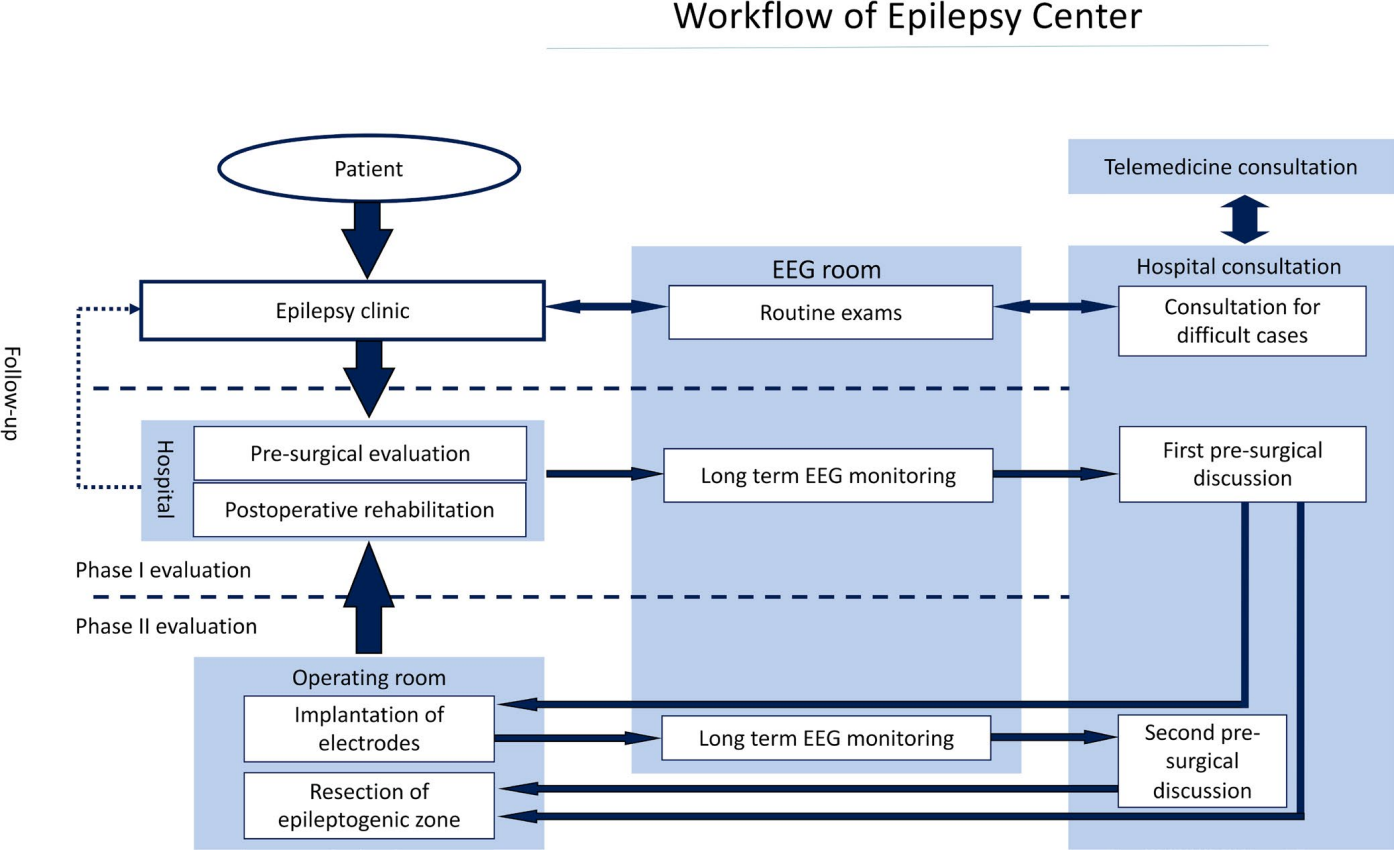
Workflow of Epilepsy Center. Abbreviations: EEG, electroencephalography

**FIGURE 2:**
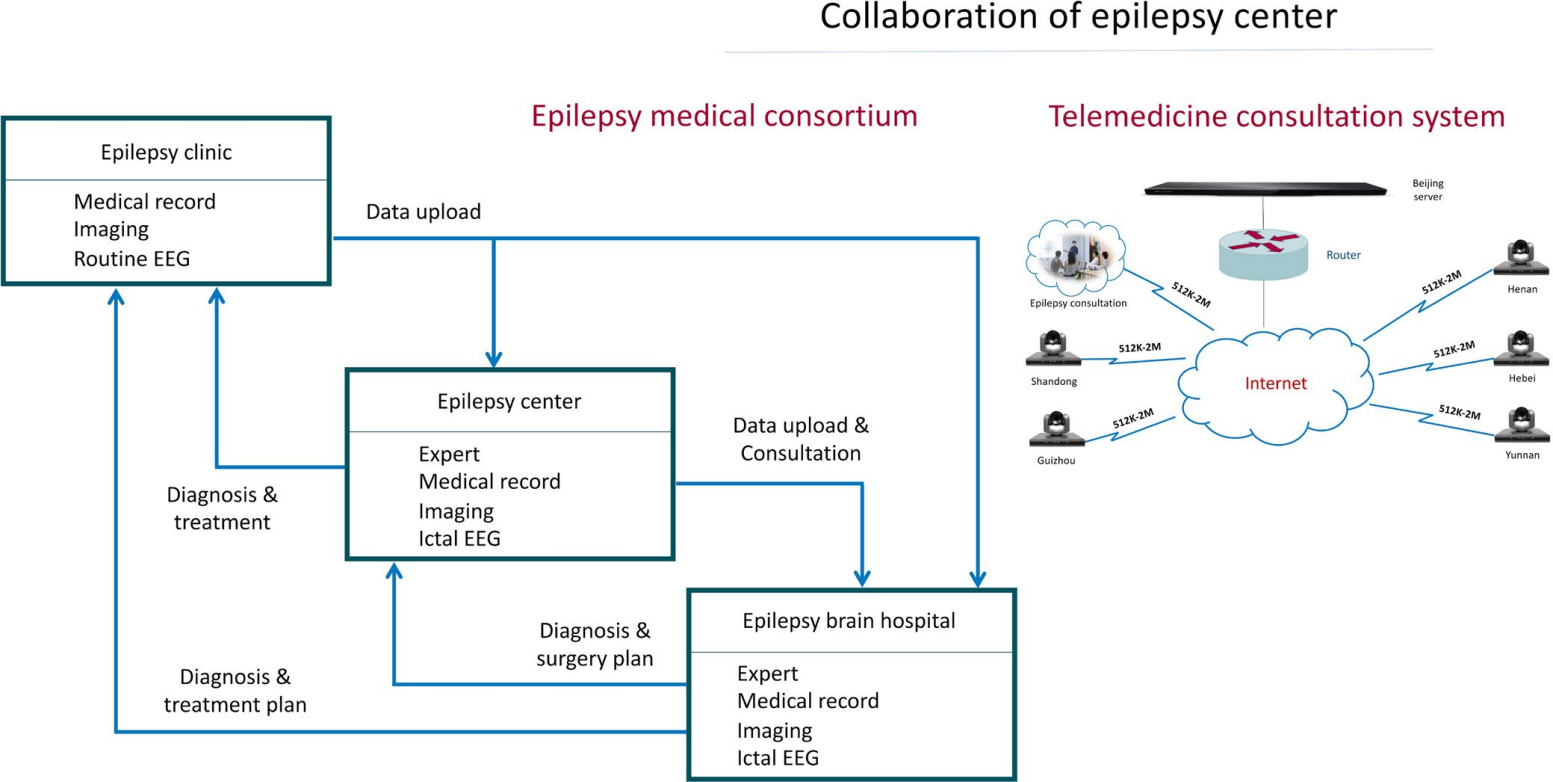
Collaboration of Epilepsy Center. Abbreviations: EEG, electroencephalography

Figures 1 and 2 show the model of the workflow of an epilepsy center and the collaboration among epilepsy centers.

### 4.2 Rural and urban community management projects on epilepsy caring

Grassroots' caring for epilepsy is critical in epilepsy governance because this is where PWE are first seen and treated, especially in rural and remote areas. Correct and precise diagnosis and treatment of new cases and required follow‐up of existing cases are essential responsibilities of public health workers in primary healthcare facilities and form the basis to epilepsy governance.

We responded to the “WHO's global campaign to bring epilepsy out of the shadows.” In partnership with the Ministry of Health and participating provincial health departments, WHO, ILAE, and IBE, a DP was implemented in 2002‐2004.^13^

Since 2005, the DP extended as the “epilepsy prevention and control project” in rural China. Up to now, 248 counties in 18 provinces had joined this project. Around 117 thousand people with convulsive epilepsy received anti‐seizure medications (ASMs) free of charge. The funding from the central government to this project increased from 4 million CHY in 2005 to 20.81 million CHY (3.32 m USD) in 2017. From 2022, the funding would be further doubled, and the project will extend to the whole country.^14^

**FIGURE 3:**
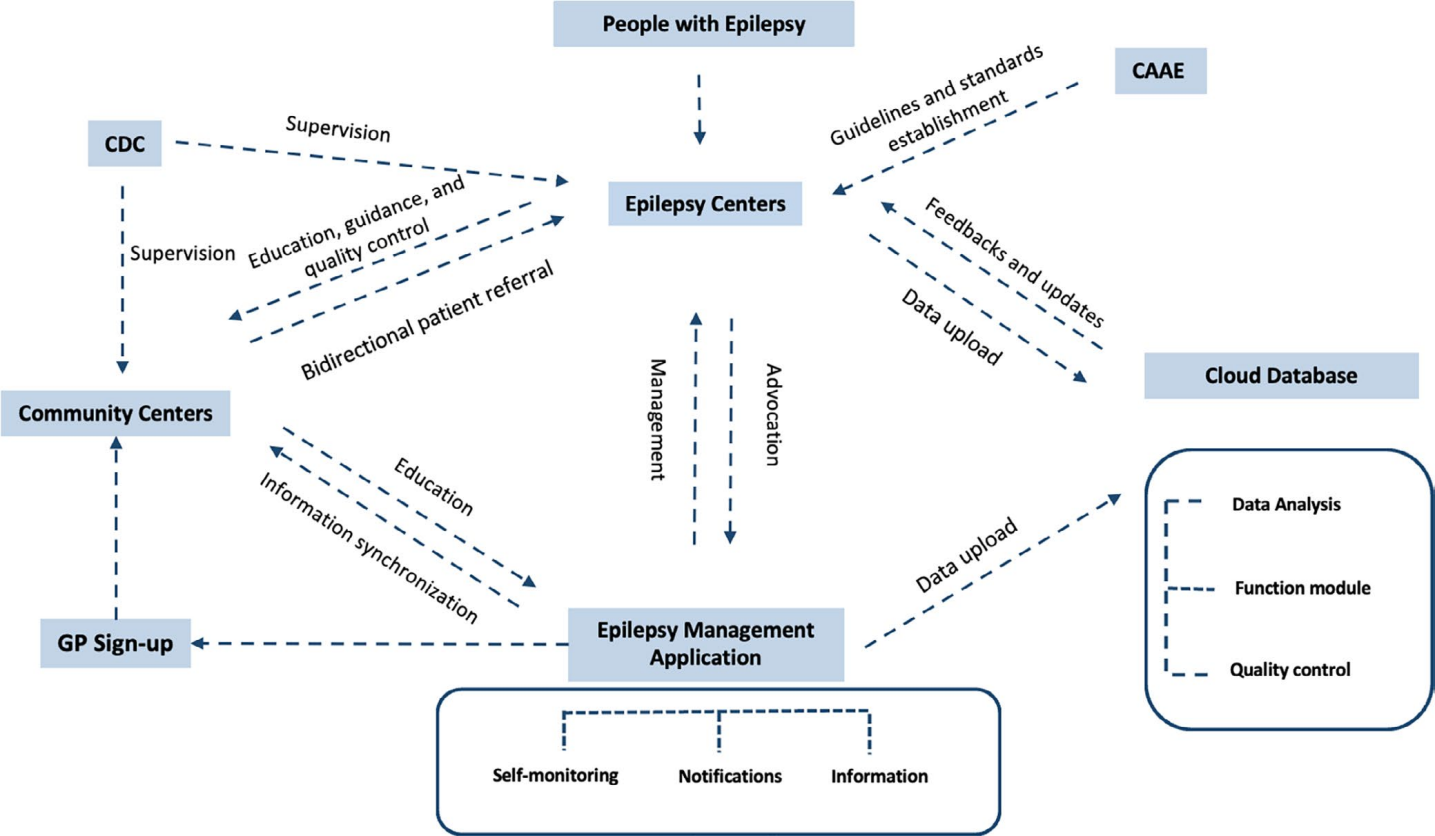
Epilepsy Urban Community Management Program. Abbreviations: CAAE, China Association Against Epilepsy; CDC, Centers for Disease Control; GP, General Practitioner

Presently, we have designed a project in urban communities and started to implement in 5 provinces in China. The working style of this project is shown in Figure 3.^15^

### 4.3 Awareness, publicity, and education

People with epilepsy and their family members are vulnerable groups in society, especially in low‐ and middle‐income countries (LMIC), as they are in China. Some traditional folk customs, misunderstandings, and lack of scientific knowledge result in stigma and discrimination towards PWE, thus preventing them from seeking appropriate treatment.

Strengthening of public awareness and popular science education to the people are badly needed in order to mobilize social awareness and care for PWE and their families, to protect and improve social welfare for PWE, to overcome prejudice and stigma, as well as to accelerate advances in treatment and research on epilepsy.

#### 4.3.1 International Epilepsy Caring Day

In 2007, the CAAE proposed and set up an “International Epilepsy Caring Day (IECD)” on June 28th in China. This was supported by more than 10 ILAE chapters. From 2007 to 2021, there have been 15 IECDs' activities in China and partly in Hong Kong. Each IECD has had a theme and a poster to be distributed nationwide; the taglines of each IECD are shown in Table 2.

**TABLE 2:** The taglines of the International Epilepsy Caring Day (June 28th, 2007–2021)

Year	Theme
2007	Reduce stigma, Out of shadows
2008	Dignity to rebuild, Society to contribute
2009	Caring patients, Guiding treatment
2010	Children with epilepsy, health, and development
2011	Follow guidelines, Say no to false advertisement
2012	Stand up for epilepsy
2013	Realize the dream to cure epilepsy, Start from compliance and diary
2014	Joint efforts by doctor & patient to defeat epilepsy
2015	Wholehearted care for a sound body and mind
2016	Ginkgo leaf logo warming your life with epilepsy
2017	Caring for school students with epilepsy
2018	Epilepsy and brain science: Epilepsy opens a door to explore the secret of human brain
2019	Epilepsy and public health
2020	Epilepsy Care in 5G Era
2021	Epilepsy care in the community

The IECD's activities were carried out nationwide every year with a total of about 60 thousand person‐times' of medical professionals, volunteers, PWE, and their family members were involved. The IECD achieved the special attention of the different government levels and mass media, so to promote epilepsy prevention and control. The most important achievement is a greater number of PWE have learnt where they can get scientific information and regular treatment of their disease. From the assessment of epilepsy centers in 2017‐2018,^10^ we can see that the annual number of outpatient visits was 1 725 170 in the 358 epilepsy centers, which is almost three times of that in 2010.^16^

#### 4.3.2 China Association Against Epilepsy Bureau for Epilepsy (CBE)

The CBE was established in 2008, firstly in Shanghai as the “Haima (seahorse) Club” and then gradually developed in other provinces and municipalities based on local “epilepsy centers.” The CBE is responsible for organizing PWE and their carers' activities and the special website to give publicity to epilepsy awareness and popular science education.

The CBE's activities included voluntary consultation, Q&A, by doctors to PWE and their carers; communication between PWE and family members to exchange experiences of self‐care; health‐promoting activities of children with epilepsy such as painting, photography, outings, summer camps, etc; encouragement of PWE and family members to give suggestions/criticism to hospital services; and reporting PWE’s needs and requirements to government, etc

#### 4.3.3 Publications for popular science education and social interests

The CAAE has published around 10 books since it established, such as the Green Book on prevention and control of epilepsy, Blue Book on financial burden of PWE, Knowledge on epilepsy, “EPILEPSY across the SPECTRUM” (Chinese translation), etc

#### 4.3.4 “Going West” project

Following the strategy of “Improving development of the western regions” called by the central government, a “Going West” project was carried out by the CAAE Youth Commission (CYC). Since 2013, teams composed of the CYC members working in tertiary hospitals in big cities were going to the “western region” – less economically developed regions of China each year. The main activities included academic and technical training lectures, grand round teaching, and personal training in out‐patient department and on‐site free of charge patient consultations. At the same time, the team had fully communication with the local government and mass media to promote effective public education and improve the government's political commitment to epilepsy control and public awareness. As of 2020, 51 counties in 25 provinces have been visited. Free of charge consultations for around 21 300 PWE and training of more than 11 900 medical personnel have been implemented in those regions visited.^3^

### 4.4 Big data platform

The benefits of big data to the medical system can predominantly be seen through strengthened management, reduction in cost and improvement of efficiency, early warnings on health, and improved decision‐making, etc. These could be the same for epilepsy management.

In China, large datasets exist from different sources, eg, the project of prevention and control of epilepsy in rural China, which has data on more than 100 000 PWE; epidemiological data from areas of some provinces; clinical data from all epilepsy centers, etc These datasets can make a solid base for an epilepsy big data platform.

Until now, however, we have not yet had a national platform for collecting, analyzing, and utilizing this tremendous amount of information. There are several reasons, including insufficient awareness on the importance of building the big data platform, lack of funds and equipment, selfish departmentalism which prevents sharing information with others, not yet having regulation on how to use the collective information by individual units or persons, etc

The upcoming “Epilepsy Centers’ Consortium” would give us a good opportunity to set up a national “Big data platform” in the not‐too‐distant future.

### 4.5 Development of telemedicine

Telemedicine is defined as the “use of advanced telecommunication technologies to exchange health information and provide healthcare services across geographic, time, social and cultural barriers.”^17^

As of March 2020, there were 904 million netizens in China,^18^ and 5G networks are available in almost every county. This fact presents a great opportunity to improve epilepsy care through telemedicine. Although the technology has already been implemented in some tertiary epilepsy centers in China, it is necessary to further design and develop them widely, especially in the rural and remote area.

### 4.6 Team construction and capacity building

The current rapid development of ASMs, epilepsy surgery, neuromodulation, and diagnostic equipment has been accompanied by an increasing interest in epilepsy among neurologists, neurosurgeons, and pediatric neurologists, who have joined the professional team of clinical epileptology. This constitutes a remarkable development and progress in the past two decades in China. A comparison of the personnel in epilepsy centers in 2010 and 2018 is shown in Table 3.^10^, ^16^

**TABLE 3:** Personnel in epilepsy centers

Epilepsy Specialists	2010^18^	2018^10^
Neurologists	2436	4103
Neurosurgeons	969	2159
Pediatric Neurologists	854	3426
Psychologists, Psychiatrists	351	368
Electro‐neurophysiologists	920	1609

In addition to the training courses in the annual national professional conferences, we have made great efforts to strengthen team capacity building.

#### 4.6.1 Training and proficiency testing of epilepsy specialists

In China, legal certification for medical specialists, including epileptologists, does not yet exist. However, there is a need to evaluate the quality of epileptologist's training at epilepsy centers with different levels of certification. In accordance with the ILAE Educational Curriculum,^19^ we organized a 5‐month online training course in 2020 and a proficiency test for level 1 (entry) and level 2 (proficiency) in epileptology. Of 773 trainees that participated, 87 passed level 1 and 505 passed level 2. This training and proficiency testing will continue in the years to come.^20^

#### 4.6.2 Training and proficiency testing of electroencephalography (EEG) physicians and technologists

Starting in 2013, and in collaboration with the National Medical Examination Center, the CAAE has organized an annual EEG training course and proficiency testing, which includes a 6‐month online training, a 3‐day face‐to‐face course and a computerized examination. From 2013 to 2020, a total of 2359 individuals have joined this program and 1888 (80%) have passed various tests as follows: 850 (36%) passed the entry level, 978 (41.5%) passed the proficiency level, and 60 (2.5%) passed the advanced level.^20^

#### 4.6.3 China Association Against Epilepsy (CAAE) video live room

Founded in 2020, the CAAE Video Live Room broadcast press releases on CAAE activities related to epilepsy care, professional lectures and virtual meetings, debate, and analysis of patients with drug‐resistant epilepsy, quizzes on epilepsy scientific knowledge, etc These programs have become quite popular among team members and have helped raise their clinical practice skills in epilepsy.

### 4.7 Strengthening epilepsy research

The 21st century has been deemed the “Century of Brain Science.” Research on epilepsy provides a special and even unique window to explore the complex functions of the human brain. Modern research in epilepsy integrates many aspects of brain structure and function, including electrical functioning, chemical signal transmission, ion channels, neuronal networks, and magnetic imaging of various types. This provides a great opportunity to find new treatments for epilepsy as well as to unlock the mysteries of the human brain.^2^

We emphasize the following topics in clinical and basic research: the epilepsy and brain science, epilepsy and public health, precision medicine in epilepsy therapeutics including genetic research, application of the brain–computer interface (BCI) and other artificial intelligence (AI) techniques.

In recent years, the central government of China increased the financial support for epilepsy research through the National Natural Science Foundation and Project 863, which focuses on high technology studies, and Project 973, which focuses on key national basic studies.

### 4.8 Developing and strengthening partnerships

In addition to the CAAE and its branches, other related professional organizations (partners) which deal with PWE in China include the sub‐units of different branches of the Chinese Medical Association (CMA), such as the Epilepsy & EEG Group of the Neurology Branch, the Functional Neurosurgery Group of the Neurosurgery Branch, the Pediatric Neurology Group of the Pediatrics Branch, and sub‐units of the Chinese Medical Doctor Association (CMDA).

Most of the board members of the CAAE are also members of those partner organizations, and many occupy leadership positions. This facilitates concerted action and collaborations in epilepsy‐related activities, such as creation of the clinical guidelines for epilepsy diagnosis and treatment, epilepsy surgery and neuromodulation norms, consensus on ASM treatment, etc

An important question that arises in partnerships is how to deal with the relationship with the pharmaceutical companies. The CAAE has had three principles for collaboration with the pharmaceutical companies: (i) not taking part directly in their advertisement and commercial activities; (ii) requesting them to fulfill their social responsibility; and (iii) allowing them to introduce their products based on scientific evidence while providing equal opportunities to other companies’.

### 4.9 Coordination with government

The CAAE is registered in the Ministry of Civil Affairs (MCA) as an independent social organization and is supervised by the NHC. Our major professional activities are approved and supported by the NHC, as the rural and urban community projects, standardization of epilepsy centers, setting up the EEG and neurophysiology technical title series, EEG training course and proficiency tests, going west project, and others. Maintaining close communication and coordination with different levels of government is an essential task of the CAAE and its branches.

### 4.10 International collaboration

The CAAE was accepted as the national chapter of the ILAE and IBE in the 26th International Epilepsy Congress held in Paris in 2005. Since then, members of the CAAE have actively participated in most of the congresses, and have worked as members of special commissions, task forces, journal editorial boards, and other public activities of the ILAE and IBE. Currently, approximately 20 experts from the CAAE serve as members of the ILAE/IBE Executive Committee, co‐editor‐in‐chief of the journal “Epilepsia Open,” and as members or co‐chairs of commissions and task forces.

Bilateral international collaboration and exchange is active as well. The CAAE has established many partnerships with institutes and organizations, such as the American Epilepsy Society (AES), national epilepsy centers of Switzerland, Germany, France, Netherlands, Italy, and others.

These multi‐lateral communications, collaborations, and professional exchange are very helpful for the success of the CAAE's efforts in epilepsy care in China.

**FIGURE 4:**
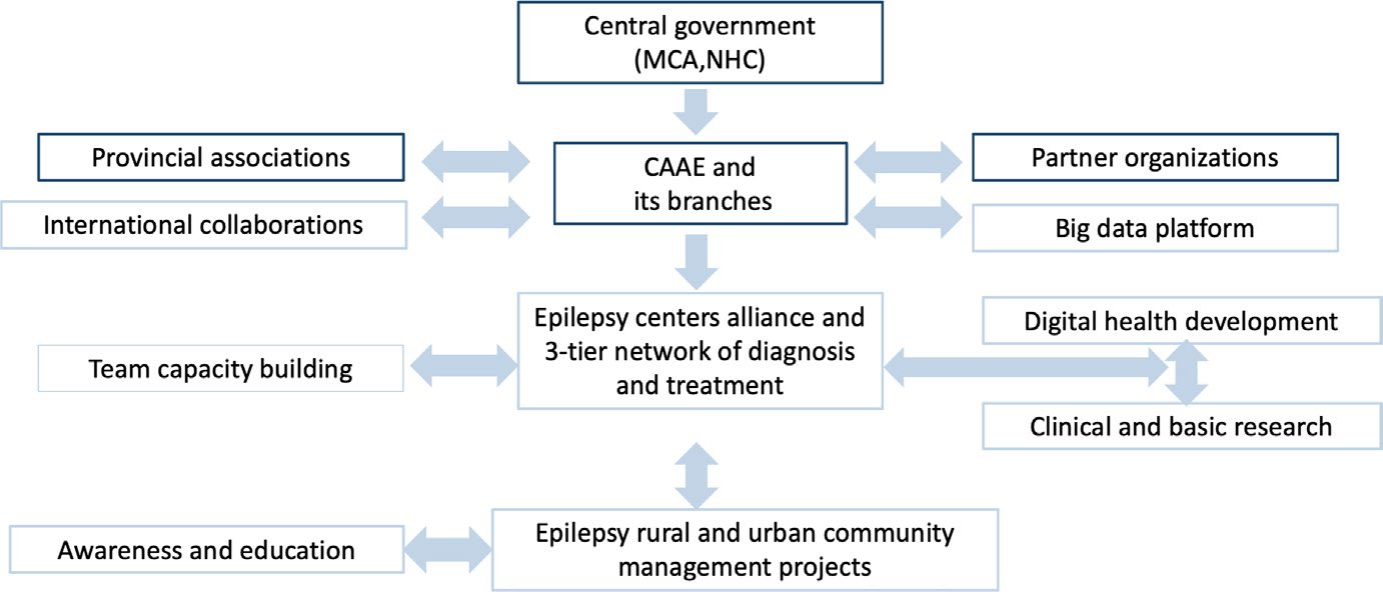
The Pattern of Governance of Epilepsy Control in China. Abbreviations: CAAE, China Association Against Epilepsy; MCA, Ministry of Civil Affairs; NHC, National Health Commission

In summary, the comprehensive governance of epilepsy prevention and control in China may be summarized in Figure 4. Among the 10 essential elements, except for the “Consortium of Epilepsy Centers” and the “Big data platform,” which are in preparatory stage, all the others are what we have done and are practicing.

## 5 CONCLUSION

Reducing the burden of epilepsy requires strong leadership and the commitment of a range of stakeholders. It requires a commitment from governments at the local, regional, national, and international level to develop strategic policy frameworks that recognize the needs of people living with epilepsy. It requires changes in health policies, plans and protective legislation, adequate funding, and good‐quality data.^1^

This article uses “the comprehensive governance of epilepsy prevention and control in China” as an example of the strategic working style for the prevention and control of epilepsy at a national level. We hope that our experience provides a useful blueprint for other LMIC. However, which part and what extent of this model of governance could be adopted by other countries must be based on their practical situation and the feasibility and operationality in their country.

## CONFLICT OF INTERESTS

None of the authors has any conflict of interest to disclose. The authors confirm that we have read the Journal's position on issues involved in ethical publication and affirm that this report is consistent with those guidelines.
